# Olfactory Regeneration with Nasally Administered Murine Adipose-Derived Stem Cells in Olfactory Epithelium Damaged Mice

**DOI:** 10.3390/cells12050765

**Published:** 2023-02-27

**Authors:** Tomoko Ishikura, Hideaki Shiga, Yuka Nakamura, Takako Kanitani, Yasuhito Ishigaki, Takaki Miwa

**Affiliations:** 1 Department of Otorhinolaryngology, Kanazawa Medical University, Kahoku 920-0293, Ishikawa, Japan; 2 Medical Research Institute, Kanazawa Medical University, Kahoku 920-0293, Ishikawa, Japan; 3 Center for Regenerative Medicine, Kanazawa Medical University Hospital, Kahoku 920-0293, Ishikawa, Japan

**Keywords:** olfactory impairment, neurotrophic factor, regenerative medicine, adipose-derived mesenchymal stem cells

## Abstract

In this study, we aimed to determine whether nasally administered murine adipose-derived stem cells (ADSCs) could support olfactory regeneration in vivo. Olfactory epithelium damage was induced in 8-week-old C57BL/6J male mice by intraperitoneal injection of methimazole. Seven days later, OriCell adipose-derived mesenchymal stem cells obtained from green fluorescent protein (GFP) transgenic C57BL/6 mice were nasally administered to the left nostril of these mice, and their innate odor aversion behavior to butyric acid was assessed. Mice showed significant recovery of odor aversion behavior, along with improved olfactory marker protein (OMP) expression on both sides of the upper-middle part of the nasal septal epithelium assessed by immunohistochemical staining 14 d after the treatment with ADSCs compared with vehicle control animals. Nerve growth factor (NGF) was detected in the ADSC culture supernatant, NGF was increased in the nasal epithelium of mice, and GFP-positive cells were observed on the surface of the left side nasal epithelium 24 h after left side nasal administration of ADSCs. The results of this study suggest that the regeneration of olfactory epithelium can be stimulated by nasally administered ADSCs secreting neurotrophic factors, thereby promoting the recovery of odor aversion behavior in vivo.

## 1. Introduction

Therapeutic approaches to improving olfactory neuron regeneration and modulating physiological olfactory dysfunction remain in pre-clinical stages [1]. Stem cells have recently attracted considerable attention given their ability to secrete several neurotrophic factors, such as nerve growth factor (NGF), insulin-like growth factor-1 (IGF-1), interleukin (IL)-15, and brain-derived neurotrophic factor (BDNF), which are important for the regeneration of olfactory sensory neurons [2,3,4,5]. Pre-clinical studies have explored the effect of intravenously administrated adipose-derived stem cells (ADSCs) [6] in rodents with damaged olfactory epithelium [7,8]. Human adipose tissue is ubiquitous and can be easily obtained in large quantities with little donor site morbidity [9]. Thus, in addition to their high secretory potential, ADSCs are superior to other types of stem cells, such as induced pluripotent stem cells or embryonic stem cells, owing to their greater availability and enhanced safety profile [10]. However, there are concerns regarding some adverse events, such as pulmonary embolism, after intravenous administration of ADSCs [11].

The basal cells of the olfactory epithelium can differentiate into olfactory sensory neurons and other supporting cells, thereby regenerating the damaged olfactory epithelium [12]. In rodents, olfactory function can be recovered 4–6 weeks after methimazole injection, which is known to specifically damage cells in the olfactory epithelium [13]. In the present study, we aimed to investigate whether nasally administered murine ADSCs could promote the recovery of odor aversion behavior and regeneration of the olfactory epithelium in mice with methimazole-induced olfactory damage.

## 2. Materials and Methods

### 2.1. Animals

Eight-week-old male C57BL/6J mice (Sankyo Labo Service Corporation, Tokyo, Japan) were housed at a constant temperature (22–26 °C) and under a 12 h light/dark cycle. Standard solid food (Sankyo Labo Service Corporation) and water were provided ad libitum. The average weight of the mice was approximately 22–23 g at the start of the experiments.

### 2.2. Methimazole Administration

It has been reported that methimazole induces apoptotic cell death in rodent olfactory neurons [14]. Therefore, to induce damage to the olfactory epithelium, the mice were intraperitoneally injected with methimazole (75 mg/kg; FUJIFILM Wako Pure Chemical Corporation, Osaka, Japan) dissolved in 0.9% saline, after the assessment of innate odor aversion behavior to butyric acid as described below, 7 days before nasal administration of ADSCs or vehicle control.

### 2.3. Nasal Administration of GFP-Positive Murine ADSCs

OriCell ADSCs derived from the adipose tissue of qualified green fluorescent protein (GFP) transgenic C57BL/6 mouse inguen were purchased from MUBMD-01101 (Cyagen Biosciences, Santa Clara, CA, USA). These cells are positive for CD44, CD90, and CD29 (>70%) and negative for CD34, CD11b, and CD45 (<5%), as determined by flow cytometry and described in the user manual. Briefly, 1 × 10^4^ OriCell ADSCs (8th passage) resuspended in 10 μL phosphate-buffered saline (PBS) were administrated in the left nasal cavity of mice using a microinjection pipette. Mice nasally administrated with 10 μL PBS alone were used as control. Sneezing was prevented with intraperitoneal administration of a mixed solution of medetomidine (0.75 μg/g), midazolam (4 μg/g), and butorphanol (5 μg/g) in PBS. Mice were awakened with intraperitoneal administration of atipamezole 30 min after nasal administration of the solution. ADSCs or PBS were nasally administered into only left nostril of each mouse to mitigate breathing difficulties after nasal administration, after the assessment of innate odor aversion behavior to butyric acid as described below.

### 2.4. Assessment of Innate Odor Aversion Behavior to Butyric Acid in Mice

Odor aversion behavior to butyric acid was assessed by determining the time the mice spent in the transparent part of a box with two compartments under a dark infrared camera covered with a plastic shading bag for the duration of the experiment (3 min) (Figure S1A). A cotton ball embedded with 300 μL 10% butyric acid in distilled water was placed inside an Eppendorf tube and attached to the closed part of the box (Figure S1B, arrow). The mice were separately familiarized with the box and its closed sections for 3 min without butyric acid before the experiment. The experimental procedures are described in Figure S1C. The mice underwent perfusion fixation as described below after assessing the innate odor aversion behavior to butyric acid on day 14.

### 2.5. Immunohistochemical Analysis of Olfactory Epithelial Cells and ADSCs

The mice were perfused with physiological saline from the left ventricle and fixed with 4% paraformaldehyde (FUJIFILM Wako, Osaka, Japan) under anesthesia, which was administered intraperitoneally as a mixed solution of medetomidine (0.75 μg/g), midazolam (4 μg/g), and butorphanol (5 μg/g). The head was then dissected, and facial bones were removed. Following overnight fixation in 4% paraformaldehyde at 4 °C, the nasal turbinates were resected and decalcified for 7 d at 4 °C with a commercial decalcification solution (KC-X; FALMA, Tokyo, Japan) at 50% dilution in PBS before paraffin embedding. Whole head samples were embedded in paraffin and cut into 3 µm coronal sections at the level of the anterior edge of the olfactory bulb, and 300 µm before and after that position, before being mounted onto slides for immunohistochemical staining.

Sections of the nasal turbinates were deparaffinized with xylene and rehydrated using a graded alcohol series. Deparaffinized sections were boiled in Tris-EDTA (pH 9.0) for 10–30 min at 95 °C for antigen retrieval. After blocking and antigen retrieval, the sections were incubated with primary antibodies (Table S1) for 1 h at 24 °C.

After blocking and antigen retrieval for the olfactory marker protein (OMP) and paired box 6 (PAX6), the sections were washed with phosphate buffer before being incubated with a biotin-conjugated secondary antibody for 30 min at 24 °C, followed by peroxidase detection (Histofine; NICHIREI BIOSCIENCES, Tokyo, Japan). For labeling of NGF and GFP, the sections were incubated for 30 min at 24 °C with the respective secondary antibodies (Table S2) and then washed with PBS. The sections were then incubated for 10 min at 24 °C with RNase A (10 μg/mL, NIPPON GENE, Toyama, Japan) in distilled water. After development, the sections were mounted in ProLong Glass Antifade Mountant with 4,6-diamidino-2-phenylindole (DAPI; Thermo Fisher Scientific, Waltham, MA, USA). Normal rabbit or goat serum was used as a negative control instead of the primary antibody solution. Slides were observed under a fluorescence microscope (BZ-X700; Keyence Corporation, Osaka, Japan).

The number of positively stained cells within a 300 µm basal membrane length of olfactory epithelium in each field was manually counted. The fields in the upper-middle part of the nasal septum (Part 1), the lower part of the nasal septum (Part 2), and the superior lateral turbinate (Part 3) on the left or right sides were assessed using three coronal sections with 300 µm intervals for the assessment of OMP expressions in the nasal epithelium of mice 14 d after left side nasal administration. The fields in Part 1 on the left or right sides were assessed using three coronal sections with 300 µm intervals for the assessment of OMP and PAX6 expressions in the nasal epithelium of mice 24 h, 3 d, and 7 d after left side nasal administration, and of NGF and GFP expressions in the nasal epithelium of mice 24 h, 3 d after left side nasal administration.

The middle one of three coronal sections included the anterior edge of the olfactory bulb. The average scores in the three coronal sections were calculated for each part. The histological analysis was separately performed by three investigators (TI, TK and HS) in a blinded manner for each sample image, and the average scores were used for statistical analysis.

### 2.6. ADSC Culture Supernatant Analysis by Enzyme-Linked Immunosorbent Assay (ELISA)

The supernatant of the murine ADSC cultures or control cell culture medium (KBM ADSC-1; KOHJIN BIO, Saitama, Japan) was collected 24 h after the cells were seeded and reached 70% confluency and was stored at −20 °C until use. The cells were not seeded on the dishes for collecting the supernatant of control cell culture medium. The neurotrophic factors, mature NGF, mature BDNF, IGF-1, and IL-15/IL-15R complex, were quantified using the NGF Rapid ELISA Kit (BEK-2213; Biosensis, Thebarton, Australia), Mature BDNF Rapid ELISA Kit (BEK-2211; Biosensis), IGF-1 mouse ELISA kit (EMIGF1; Invitrogen, Waltham, MA, USA), and IL-15/IL-15R complex mouse ELISA kit (BMS6023; Invitrogen), respectively, according to the manufacturers’ instructions. Duplicate ELISA assessments were performed for each well in a 96-well plate. Sample collection was repeated three times, and the medians with interquartile ranges were calculated.

### 2.7. Statistical Analysis

Median values were compared using the Mann–Whitney test. Prism 6 (GraphPad Software, San Diego, CA, USA) was used for all statistical analyses. All *p*-values are two-tailed. Data are shown as medians with interquartile ranges unless otherwise indicated. Statistical significance was set at *p* < 0.05.

## 3. Results

### 3.1. Odor Aversion Behavior Analysis in Mice with Olfactory Damage

**Figure 1:**
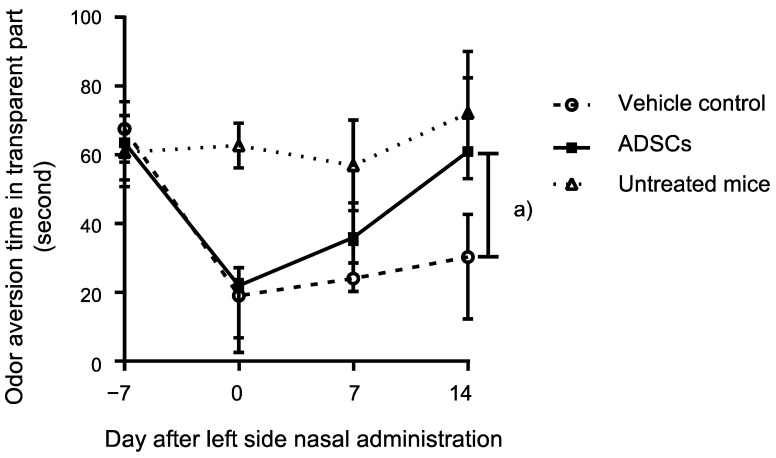
Odor aversion behavior analysis of mice treated with murine adipose-derived stem cells (ADSCs). Comparison of odor aversion behavior to butyric acid determined with located time in the transparent part of the box over 3 min of observation between ADSC- and PBS-treated olfactory-impaired mice on day 14. The points with bars indicate medians with interquartile range. The untreated mice were also assessed with odor aversion behavior to butyric acid. Statistical significance was determined by the Mann–Whitney test. (a) *p* = 0.002.

Butyric acid is frequently used to determine the innate odor aversion behavior in rodents [15]. Thus, herein, we evaluated the ability of nasally administrated ADSCs to promote the recovery of odor aversion behavior to butyric acid in mice with methimazole-induced olfactory damage. ADSC-administrated mice (*n* = 6) showed a significantly stronger response to butyric acid than the control mice (*n* = 6) 14 d after treatment (*p* = 0.002, Mann–Whitney test; Figure 1). The untreated mice with methimazole or nasal administration of ADSCs or PBS (*n* = 4) did not show a decrease of innate odor aversion behavior to butyric acid on each time point from the starting point (*p* = 0.91, *p* = 0.46, *p* = 0.26, Mann–Whitney test; 0 d, 7 d, 14 d, respectively, Figure 1). ADSC-administrated mice were not significantly different to the untreated mice for the innate odor aversion behavior to butyric acid at 14 d (*p* = 0.71, Mann–Whitney test; Figure 1). These findings indicate that nasal administration of ADSCs can support the recovery of innate odor aversion behavior upon olfactory epithelium damage to the normal level.

### 3.2. Immunohistochemical Analysis of Olfactory Marker Protein Expression in the Nasal Epithelium of Mice on Day 14 after Left Side Nasal Administration of ADSCs

**Figure 2:**
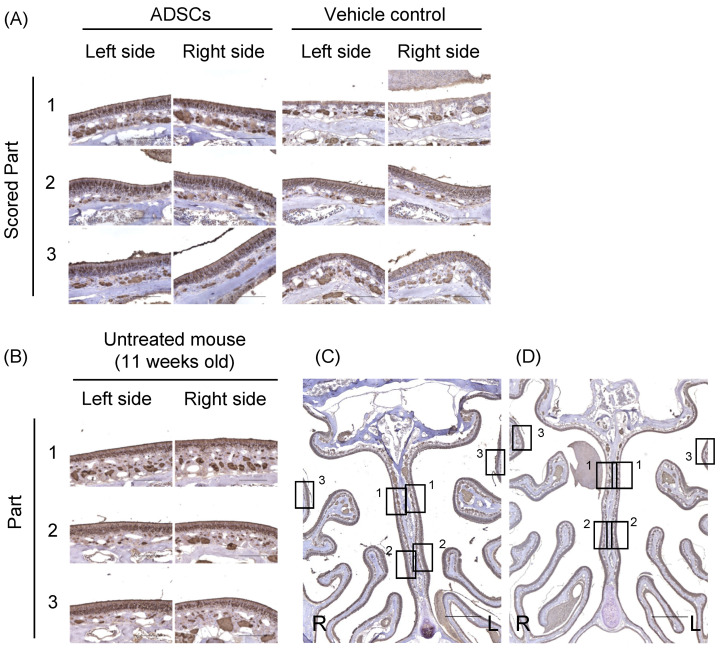
Immunohistochemical analysis of olfactory marker protein in the nasal epithelium of mice 14 d after nasal administration of ADSCs. (**A**) Expression of the olfactory marker protein (OMP) in the olfactory epithelium of mice treated with ADSCs or PBS. Scored Part 1: the left and right side of the upper-middle part of the nasal septum. Scored Part 2: the left and right side of the lower part of the nasal septum. Scored Part 3: the left and right side of the superior lateral turbinate. (**B**) The expression of OMP detected in the middle layer of the nasal septum and turbinate epithelium in an untreated 11-week-old male C57BL/6J mouse. (**C**,**D**) Squares indicate the parts of an assessment in the lower power field image in the olfactory epithelium of mice treated with ADSCs (**C**) or PBS (**D**). Images are representative of six mice for each treatment group (**A**,**C**,**D**), and representative of four untreated mice (**B**). Bars = 100 μm (**A**,**B**) and 500 μm (**C**,**D**).

The olfactory marker protein (OMP) is exclusively expressed in mature olfactory receptor neurons [16]. Immunohistochemical analysis showed that the expression levels of OMP in the left and right sides of the upper-middle part of the nasal septum (Part 1) were significantly increased in ADSC-treated mice (*n* = 6) compared to controls (*n* = 6) (Left side, *p* = 0.002; Right side, *p* = 0.002; Mann–Whitney test; Table 1, Figure 2A and Figure S2). On the other side, the expression levels of OMP in the left and right sides of the lower part of the nasal septum (Part 2) and on the superior lateral turbinate (Part 3) were not significantly different between ADSC-treated mice and controls (Table 1, Figure 2A and Figure S2). Furthermore, we compared the expression of OMP between the left and right sides of the upper-middle part of the nasal septum in ADSC-treated mice. The expression of OMP was not significantly different between the left (*n* = 6) and right sides (*n* = 6) (*p* = 0.79, Mann–Whitney test). The expression of OMP was detected in the middle layer of the nasal septum and turbinate epithelium in the untreated mouse (Figure 2B). These results suggest that nasal administration of ADSCs promotes the regeneration of mature olfactory neurons in both sides of the epithelium of the upper-middle part of the nasal septum in mice 14 d after left side nasal administration.

### 3.3. Immunohistochemical Analysis of the Olfactory Epithelium in Mice 24 h, 3 d, and 7 d after Left Side Nasal Administration of ADSCs

**Figure 3:**
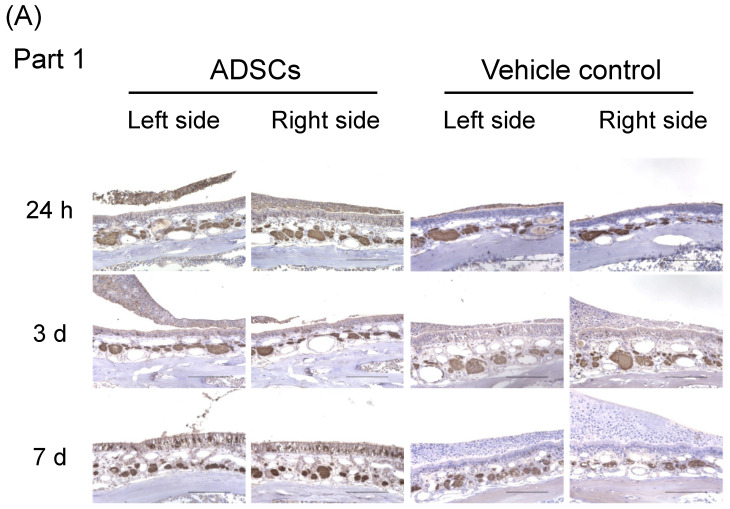

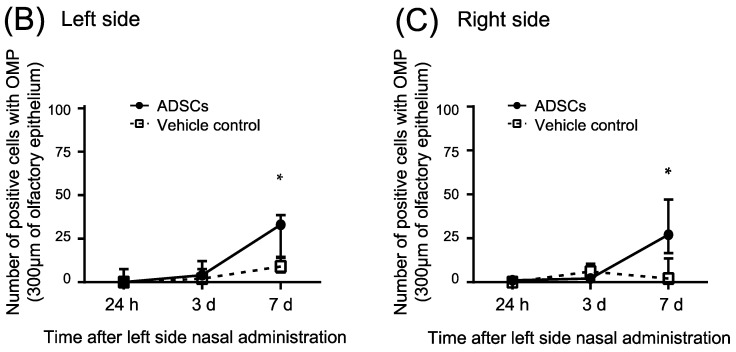
Immunohistochemical analysis of olfactory marker protein in the upper-middle region of the nasal septum (Part 1) of mice 24 h, 3 d, and 7 d after nasal administration of ADSCs. (**A**) Representative images of immunohistochemical staining of olfactory marker protein (OMP). (**B**,**C**) Number of positive cells with OMP in the upper-middle region of the nasal septum of mice. (**B**) Left side. (**C**) Right side. Images are representative of five mice for each treatment group. Bars = 100 μm. Statistical significance was determined by the Mann–Whitney test. * *p* < 0.05.

For a better understanding of the underlying mechanisms triggered by ADSCs on the regeneration of olfactory epithelium in the upper-middle part of the nasal septum (Part 1), we conducted additional immunohistochemical analyses 24 h, 3 d, and 7 d after the cells were nasally administrated (Figure S3). OMP expression was low in the nasal epithelium 24 h and 3 d after nasal administration of ADSCs or PBS (Table 2, Figure 3A–C and Figure S4). Expression of OMP significantly increased in the nasal epithelium 7 d after nasal administration of ADSCs (*n* = 5) as compared with that in the controls (*n* = 5) (Table 2, Figure 3A–C; Left side, *p* = 0.032 and Right side, *p* = 0.016, respectively, Mann–Whitney test). These results suggest that nasal administration of ADSCs promotes the regeneration of mature olfactory neurons in mice 7 d after nasal administration before the recovery of odor aversion behavior.

**Figure 4:**
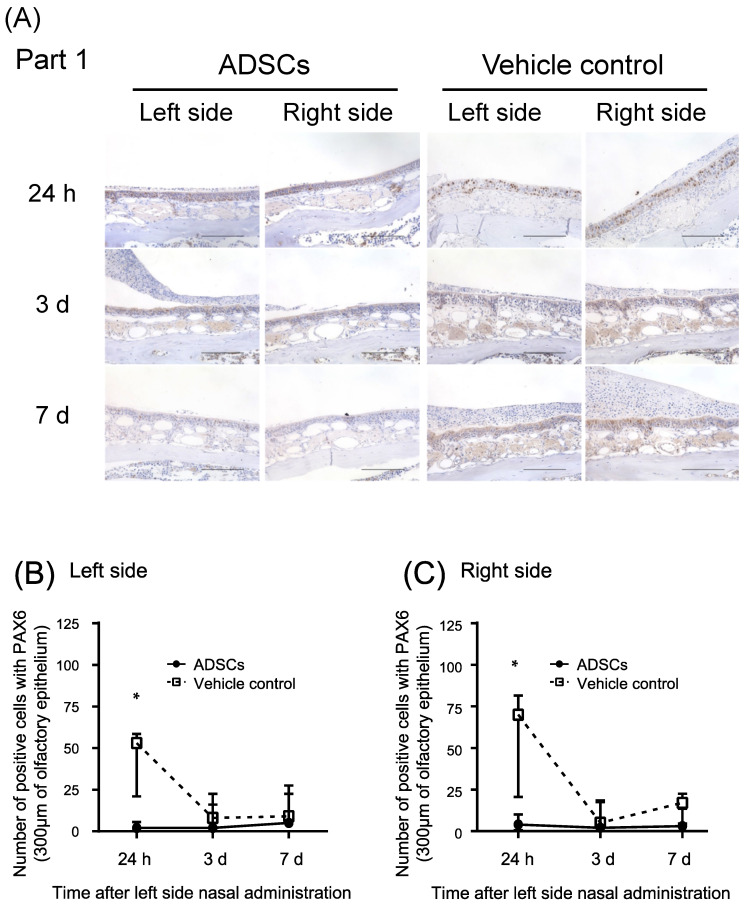
Immunohistochemical analysis of paired box 6 in the upper-middle region of the nasal septum (Part 1) of mice 24 h, 3 d, and 7 d after left side nasal administration of ADSCs. (**A**) Representative images of immunohistochemical staining of paired box 6 (PAX6). (**B**,**C**) Number of positive cells with PAX6 in the upper-middle region of the nasal septum of mice. (**B**) Left side. (**C**) Right side. Images are representative of five mice for each treatment group. Bars = 100 μm. Statistical significance was determined by the Mann–Whitney test. * *p* < 0.05.

Conversely, PAX6 expression in globose basal cells during the recovery of olfactory epithelium [17] significantly decreased in the nasal epithelium 24 h after nasal administration of ADSCs (*n* = 5) as compared with that in the controls (*n* = 5) (Table 3, Figure 4A–C and Figure S5; Left side, *p* = 0.008 and Right side, *p* = 0.008, respectively, Mann–Whitney test). PAX6 expression was low in the nasal epithelium 3 d and 7 d after nasal administration of ADSCs or PBS (Table 3, Figure 4A–C and Figure S5).

These results suggest that nasal administration of ADSCs promotes the differentiation of globose basal cells to olfactory neurons in both sides of the epithelium of the upper-middle part of the nasal septum in mice after left side nasal administration.

### 3.4. Neurotrophic Factors in ADSC Culture Medium Supernatant

To determine whether our murine ADSCs were able to secrete neurotrophic factors in 24 h, we quantified the amount of mature NGF, mature BDNF, IGF1, and IL-15/IL-15R complex present in the supernatant of the cell culture medium by ELISA. Mature NGF was detected at high levels in the supernatant of the ADSC culture medium, whereas it was below the detection limit in the KBM ADSC-1 control medium (Table 4). A small difference was observed in the mature BNDF levels between the supernatants of the ADSC and control culture media (Table 4). IGF-1 and IL-15/IL-15R were not detectable in both ADSC and control culture media (Table 4). These results indicate that murine ADSCs secrete neurotrophic factors, such as NGF in vitro.

### 3.5. Immunohistochemical Analysis of Nerve Growth Factor Expression in Olfactory Epithelium with GFP-Positive ADSCs Adhering to the Surface of Nasal Epithelium in Mice after Left Side Nasal Administration of ADSCs

**Figure 5:**
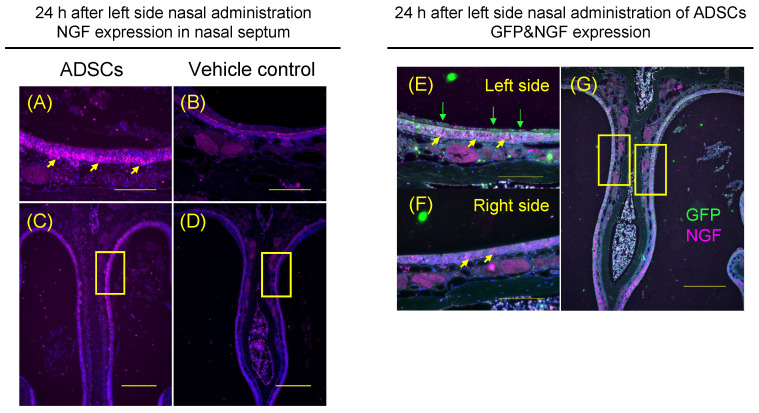
Expression of nerve growth factor by ADSCs in vivo. (**A**–**D**) Representative images of immunohistochemical staining of nerve growth factor (NGF) in the five mice after nasal administration of ADSCs (**A**,**C**) or in the mice after nasal administration of PBS (**B**,**D**). Arrows indicate NGF (**A**). (**E**–**G**) Immunohistochemical analysis of ADSCs by GFP signal (Green) and NGF expression (Purple) in the nasal cavity and epithelium of ADSC-treated mice. Images are representative of five mice treated with ADSCs and of five mice treated with PBS. Yellow arrows indicate NGF and green arrows indicate GFP (**E**). Arrows indicate NGF (**F**). Bars = 100 μm (**A**,**B**,**E**,**F**) and 250 μm (**C**,**D**,**G**).

To determine whether the increased recovery of olfactory epithelium was accompanied by increased NGF expression in the nasal epithelium, the expression of NGF was assessed in the nasal epithelium of ADSC-treated mice and the controls. The levels of NGF were significantly increased in the left side upper-middle part of nasal septum in ADSC-treated mice (*n* = 5) 24 h after nasal administration of ADSCs compared with the controls (*n* = 5) (Figure 5A–D; *p* = 0.002, Mann–Whitney test).

Furthermore, to determine whether the ADSCs expressed NGF in the nasal cavity of mice, we assessed the co-expression of GFP and NGF with double staining of these markers in ADSC-treated mice (*n* = 5). GFP was expressed on the left-side surface of nasal septal epithelium (Figure 5E), not on the right-side surface of the nasal septal epithelium (Figure 5F). GFP was not expressed in the left and right sides of nasal septal epithelium in the mice (Figure 5E,F). NGF was expressed on the left and right sides of the nasal septal epithelium of mice (Figure 5E,F). However, the levels of NGF were significantly increased in the left side of the upper-middle part of nasal septum compared with the right side of that in ADSC-treated mice (*n* = 5) 24 h after nasal administration of ADSCs (Figure 5E,F; *p* = 0.002, Mann–Whitney test). Thus, ADSCs nasally administered into the left side of the nasal cavity can increase NGF expression on the left side of the nasal septal epithelium more than the right side of the nasal septal epithelium in the mice.

Additionally, we determined whether the nasally administered ADSCs were expressed 3 d after nasal administration of ADSCs. GFP was not observed in the nasal cavities and nasal epithelium of mice 3 d after nasal administration of ADSCs (Figure S6A–C). These results indicate that nasally administered ADSCs were cleared from the nasal cavities from 24 h to 3 d after administration.

## 4. Discussion

Previous studies have demonstrated that systemic administration of rodent or human ADSCs can induce olfactory epithelium regeneration in rodents [7,8]. However, systemic administration of ADSCs can also cause adverse events, such as pulmonary embolism [11]. Therefore, clinical trials on systemic administration of ADSCs have not been performed in patients with olfactory dysfunction. Herein, we provide pre-clinical evidence that nasally administered ADSCs can support olfactory regeneration in vivo. Our results showed enhanced recovery of odor aversion behavior and olfactory epithelium regeneration in mice with damaged olfactory epithelium after treatment with nasally administered murine ADSCs, suggesting that topical implantation of autologous ADSCs may be a plausible treatment for sensorineural olfactory dysfunction.

ADSCs were observed in the nasal cavity of mice 24 h after their administration; however, after 3 d, they were cleared from the nasal cavity. On the contrary, ADSCs were not integrated into the nasal epithelium of mice 24 h after their administration. These results support a hypothesis for the safety of topical implantation of autologous ADSCs in future clinical trials.

Notably, the number of globose basal cells expressing PAX6 decreased in the olfactory epithelium of mice after 24 h, and the number of mature olfactory neurons expressing OMP started to increase 7 d after nasal administration of transgenic murine ADSCs. Those results suggest the hypothesis that nasally administered ADSCs can facilitate the differentiation of globose basal cells in vivo. Furthermore, our murine ADSCs secreted neurotrophic factors, such as NGF in vitro, and the expression of NGF was significantly increased in the nasal epithelium of ADSC-treated mice. These results are consistent with those of previous reports, which showed that NGF promotes cell differentiation during regeneration of the olfactory epithelium in rodents [18]. We did not determine whether the expression levels of BDNF were increased in the nasal epithelium of mice because a small difference in the mature BDNF levels was observed between ADSC and control culture media in vitro.

The expression of NGF in the olfactory bulb has been shown to be increased in mice challenged with methimazole [19]. Moreover, regeneration of the olfactory epithelium has been reported to be impaired in mice treated with an antibody that prevented NGF expression in olfactory bulbs after the transection of olfactory sensory neurons [20]. Furthermore, NGF in olfactory bulbs has been shown to be transported to the olfactory epithelium of mice in a retrograde fashion [18]. These findings are consistent with our results, which show that topical administration of ADSCs secreting NGF is valuable for the regeneration of the olfactory epithelium in vivo. Notably, NGF has been reported to have nutritional and regenerative properties [21]. Indeed, the safety and usefulness of nasal administration of NGF-based treatments were described in a child with severe traumatic brain injury [22]. Conversely, systemic administration of NGF causes adverse events such as neuropathic pain [23]. Furthermore, the elimination half-life of NGF was reported to be 2.3 h after intravenous injection in mice [24], whereas its plasma half-life was only 7.2 min in vivo [25]. Our results showed that nasal administration of ADSCs might not only represent a valuable source of NGF but also ensure its action for more than 24 h in the nasal cavity of mice. As murine ADSCs were not detected in the olfactory epithelium or nasal cavity 3 d after administration, they are unlikely to undergo malignant transformation.

The levels of NGF were significantly increased in the left side of the upper-middle part of nasal septum compared with the right side of that in ADSC-treated mice 24 h after left side nasal administration of ADSCs. On the contrary, the expression of PAX6 was not significantly different between the left and right sides in ADSC-treated mice 24 h after left side nasal administration of ADSCs. These results suggest a hypothesis that the amount of NGF in the left side of the upper-middle part of nasal septum secreted by our murine ADSCs on the left-side surface of nasal septal epithelium were more than that required to promote globose basal cell differentiation during regeneration of the olfactory epithelium.

In this study, a part of the neurotrophic factors such as NGF secreted by our murine ADSCs in the left side nasal cavity of mice may migrate to the opposite side through the nasopharynx, because the regeneration was increased in both sides of the upper-middle part of nasal septum. The unilateral administration of neurotrophic factors in gelatin hydrogel increases regeneration of the damaged olfactory epithelium in both sides of nasal septum in mice [26].

Further investigations are required to determine whether the expression of NGF or other neurotrophic factors by ADSCs from healthy volunteers is also correlated with the regeneration of the olfactory epithelium in vivo before starting clinical trials on topical autologous ADSC transplantation in patients with olfactory impairment. Moreover, we did not determine whether nasal administration of ADSC culture medium could improve the recovery of odor aversion behavior and regeneration of the olfactory epithelium in mice with olfactory epithelium damage because fibroblast growth factors were found to be at high levels in the culture medium (KBM ADSC-1) including fetal bovine serum (data not shown). Although the number of animals may not have been large enough for the behavioral analysis, the histological experiment made up for the shortage in this study.

## 5. Conclusions

Nasal administration of ADSCs, which secrete neurotrophic factors such as NGF, can support the recovery of odor aversion behavior and regeneration of the olfactory epithelium in mice with olfactory epithelium damage. These results are consistent with the hypothesis that topical implantation of ADSCs may be a plausible treatment for sensorineural olfactory dysfunction.

## Figures and Tables

**Table 1:** Immunohistochemical analysis of olfactory marker protein expression in the olfactory epithelium of mice 14 d after left side nasal administration of ADSCs or PBS.

Scored Part	ADSCs in PBS (*n* = 6)	Control (PBS) (*n* = 6)	*p*-Value (Mann–Whitney Test)
Left side 1	81.0 (60.3–99.3)	17.0 (5.8–25.5)	0.002
Left side 2	97.0 (85.0–123.5)	104.0 (58.0–161.0)	>0.999
Left side 3	87.5 (77.8–105.0)	77.0 (57.0–91.5)	0.320
Right side 1	80.0 (62.5–85.5)	9.0 (4.8–23.5)	0.002
Right side 2	106.5 (82.0–118.0)	118.0 (61.5–153.5)	0.650
Right side 3	108.5 (84.3–134.5)	71.0 (49.5–108.5)	0.120

Abbreviations: ADSCs, adipose tissue-derived stem cells; PBS, phosphate-buffered saline. The number of positive cells per 300 μm for each epithelium marker is represented as median (interquartile range). Scored parts: 1, upper-middle part of nasal septum; 2, lower part of nasal septum; 3, superior lateral turbinate.

**Table 2:** Immunohistochemical analysis of olfactory marker protein (OMP) expression in the olfactory epithelium of mice 24 h, 3 d, and 7 d after left side nasal administration of ADSCs or PBS.

Scored Part 1		ADSCs in PBS (*n* = 5)	Control (PBS) (*n* = 5)	*p*-Value (Mann–Whitney Test)
24 h	Left side	0.0 (0.0–7.5)	0.0 (0.0–2.5)	0.723
	Right side	1.0 (0.5–2.0)	0.0 (0.0–1.5)	0.370
3 d	Left side	4.0 (1.5–7.5)	2.0 (0.5–12.0)	0.952
	Right side	2.0 (1.0–4.5)	6.0 (1.5–10.5)	0.238
7 d	Left side	33.0 (14.0–38.5)	9.0 (5.5–14.5)	0.032
	Right side	27.0 (16.5–47.0)	2.0 (1.5–13.5)	0.016

Abbreviations: ADSCs, adipose tissue-derived stem cells; PBS, phosphate-buffered saline. The number of positive cells per 300 μm for each epithelium marker is represented as median (interquartile range). Scored part 1: upper-middle part of nasal septum.

**Table 3:** Immunohistochemical analysis of paired box 6 (PAX6) expression in the olfactory epithelium of mice 24 h, 3 d, and 7 d after left side nasal administration of ADSCs or PBS.

Scored Part 1		ADSCs in PBS (*n* = 5)	Control (PBS) (*n* = 5)	*p*-Value (Mann–Whitney Test)
24 h	Left side	2.0 (0.5–5.5)	53.0 (21.0–58.5)	0.008
	Right side	4.0 (0.5–10.0)	70.0 (20.5–81.5)	0.008
3 d	Left side	2.0 (0.5–22.5)	8.0 (6.0–16.0)	0.643
	Right side	2.0 (1.5–17.5)	5.0 (4.0–18.5)	0.341
7 d	Left side	5.0 (3.0–22.5)	9.0 (5.0–27.5)	0.651
	Right side	3.0 (0.5–13.5)	17.0 (4.5–22.5)	0.151

Abbreviations: ADSCs, adipose tissue-derived stem cells; PBS, phosphate-buffered saline. The number of positive cells per 300 μm for each epithelium marker is represented as median (interquartile range). Scored part 1: upper-middle part of nasal septum.

**Table 4:** Quantification of neurotrophic factors in ADSC culture supernatant with ELISA.

Marker (pg/mL)	ADSC Culture Medium (*n* = 3)	Control Culture Medium (*n* = 3)
Mature NGF	136.1 (74.7–246.8)	N.D.
Mature BDNF	17.9 (5.1–23.8)	N.D.
IGF-1	N.D.	N.D.
IL-15 & IL-15R	N.D.	N.D.

Abbreviations: BDNF, brain-derived neurotrophic factor; IGF-1, insulin-like growth factor-1; IL-15, interleukin 15; IL-15R, interleukin 15 receptor; N.D., not detected (below the detection limit); NGF, nerve growth factor. Data are represented as median (interquartile range).

## Data Availability

The data that support the findings of this study are available from the corresponding author upon reasonable request.

## Supplementary Materials

The following supporting information can be downloaded at: https://www.mdpi.com/article/10.3390/cells12050765/s1, Figure S1: Odor aversion behavior analysis of mice treated with murine adipose-derived stem cells (ADSCs); Figure S2: Immunohistochemical analysis of olfactory marker protein (OMP) expressions on the nasal septum and turbinates of mice 14 d after left side nasal administration of ADSCs or vehicle control (PBS); Figure S3: Experimental protocol: Immunohistochemical analysis of mice 24 h, 3 d, and 7 d after left side nasal administration of ADSCs or vehicle control (PBS); Figure S4: Immunohistochemical analysis of olfactory marker protein on the upper-middle region of the nasal septum of mice 24 h, 3 d, and 7 d after left side nasal administration of ADSCs or vehicle control (PBS); Figure S5: Immunohistochemical analysis of paired box 6 on the upper-middle region of the nasal septum of mice 24 h, 3 d, and 7 d after left side nasal administration of ADSCs or vehicle control (PBS); Figure S6: Immunohistochemical analysis of mice 3 d after left side nasal administration of ADSCs; Table S1: Primary antibodies used in this study; Table S2: Fluorescent secondary antibodies used in this study.

## References

[1] Goncalves S., Goldstein B.J. (2016). Pathophysiology of olfactory disorders and potential treatment strategies. Curr. Otorhinolaryngol. Rep..

[2] Miwa T., Moriizumi T., Horikawa I., Uramoto N., Ishimaru T., Nishimura T., Furukawa M. (2002). Role of nerve growth factor in the olfactory system. Microsc. Res. Tech..

[3] Scolnick J.A., Cui K., Duggan C.D., Xuan S., Yuan X.B., Efstratiadis A., Ngai J. (2008). Role of IGF signaling in olfactory sensory map formation and axon guidance. Neuron.

[4] Umehara T., Udagawa J., Takamura K., Kimura M., Ishimitsu R., Kiyono H., Kawauchi H., Otani H. (2009). Role of interleukin-15 in the development of mouse olfactory nerve. Congenit. Anom..

[5] Frontera J.L., Cervino A.S., Jungblut L.D., Paz D.A. (2015). Brain-derived neurotrophic factor (BDNF) expression in normal and regenerating olfactory epithelium of Xenopus laevis. Ann. Anat..

[6] Tomita K., Madura T., Sakai Y., Yano K., Terenghi G., Hosokawa K. (2013). Glial differentiation of human adipose-derived stem cells: Implications for cell-based transplantation therapy. Neuroscience.

[7] Kim Y.M., Choi Y.S., Choi J.W., Park Y.H., Koo B.S., Roh H.J., Rha K.S. (2009). Effects of systemic transplantation of adipose tissue-derived stem cells on olfactory epithelium regeneration. Laryngoscope.

[8] Franceschini V., Bettini S., Pifferi S., Menini A., Siciliano G., Ognio E., Brini A.T., Di Oto E., Revoltella R.P. (2014). Transplanted human adipose tissue-derived stem cells engraft and induce regeneration in mice olfactory neuroepithelium in response to dichlobenil subministration. Chem. Sens..

[9] Mizuno H., Tobita M., Uysal A.C. (2012). Concise review: Adipose-derived stem cells as a novel tool for future regenerative medicine. Stem. Cells..

[10] Kuroda Y., Kitada M., Wakao S., Nishikawa K., Tanimura Y., Makinoshima H., Goda M., Akashi H., Inutsuka A., Niwa A. (2010). Unique multipotent cells in adult human mesenchymal cell populations. Proc. Natl. Acad. Sci. USA.

[11] Jung J.W., Kwon M., Choi J.C., Shin J.W., Park I.W., Choi B.W., Kim J.Y. (2013). Familial occurrence of pulmonary embolism after intravenous, adipose tissue-derived stem cell therapy. Yonsei Med. J..

[12] Beites C.L., Kawauchi S., Crocker C.E., Calof A.L. (2005). Identification and molecular regulation of neural stem cells in the olfactory epithelium. Exp. Cell Res..

[13] Ueda T., Sakamoto T., Kobayashi M., Kuwata F., Ishikawa M., Omori K., Nakagawa T. (2019). Optical coherence tomography for observation of the olfactory epithelium in mice. Auris Nasus Larynx.

[14] Sakamoto T., Kondo K., Kashio A., Suzukawa K., Yamasoba T. (2007). Methimazole-induced cell death in rat olfactory receptor neurons occurs via apoptosis triggered through mitochondrial cytochrome c-mediated caspase-3 activation pathway. J. Neurosci. Res..

[15] Sasajima H., Miyazono S., Noguchi T., Kashiwayanagi M. (2015). Intranasal administration of rotenone in mice attenuated olfactory functions through the lesion of dopaminergic neurons in the olfactory bulb. Neurotoxicology.

[16] Buiakova O.I., Baker H., Scott J.W., Farbman A., Kream R., Grillo M., Franzen L., Richman M., Davis L.M., Abbondanzo S. (1996). Olfactory marker protein (OMP) gene deletion causes altered physiological activity of olfactory sensory neurons. Proc. Natl. Acad. Sci. USA.

[17] Guo Z., Packard A., Krolewski R.C., Harris M.T., Manglapus G.L., Schwob J.E. (2010). Expression of pax6 and sox2 in adult olfactory epithelium. J. Comp. Neurol..

[18] Miwa T., Horikawa I., Uramoto N., Ishimaru T., Yamamoto K., Furukawa M., Kato T., Moriizumi T. (1998). TrkA expression in mouse olfactory tract following axotomy of olfactory nerves. Acta Otolaryngol. Suppl..

[19] Yamada K., Shiga H., Noda T., Harita M., Ishikura T., Nakamura Y., Hatta T., Sakata-Haga H., Shimada H., Miwa T. (2020). The impact of ovariectomy on olfactory neuron regeneration in mice. Chem. Sens..

[20] Miwa T., Uramoto N., Ishimaru T., Furukawa M., Shiba K., Morjizumi T. (1998). Retrograde transport of nerve growth factor from olfactory bulb to olfactory epithelium. NeuroReport.

[21] Aloe L., Rocco M.L., Balzamino B.O., Micera A. (2015). Nerve growth factor: A focus on neuroscience and therapy. Curr. Neuropharmacol..

[22] Chiaretti A., Conti G., Falsini B., Buonsenso D., Crasti M., Manni L., Soligo M., Fantacci C., Genovese O., Calcagni M.L. (2017). Intranasal nerve growth factor administration improves cerebral functions in a child with severe traumatic brain injury: A case report. Brain Inj..

[23] Petty B.G., Cornblath D.R., Adornato B.T., Chaudhry V., Flexner C., Wachsman M., Sinicropi D., Burton L.E., Peroutka S.J. (1994). The effect of systemically administered recombinant human nerve growth factor in healthy human subjects. Ann. Neurol..

[24] Tria M.A., Fusco M., Vantini G., Mariot R. (1994). Pharmacokinetics of nerve growth factor (NGF) following different routes of administration to adult rats. Exp. Neurol..

[25] Poduslo J.F., Curran G.L. (1996). Permeability at the blood-brain and blood-nerve barriers of the neurotrophic factors: NGF, CNTF, NT-3, BDNF. Brain Res. Mol. Brain Res..

[26] Fukuda Y., Katsunuma S., Uranagase A., Nota J., Nibu K.I. (2018). Effect of intranasal administration of neurotrophic factors on regeneration of chemically degenerated olfactory epithelium in aging mice. Neuroreport.

