# Supplementary material for: Olfactory Regeneration with Nasally Administered Murine Adipose-Derived Stem Cells in Olfactory Epithelium Damaged Mice
Source: Cells. 2023 Feb 27;12(5):765. doi: 10.3390/cells12050765 (PMC10001053; doi:10.3390/cells12050765)
Supplement: Supplementary file 1 [file cells-12-00765-s001.zip › cells-2218627-supplementary.pdf]

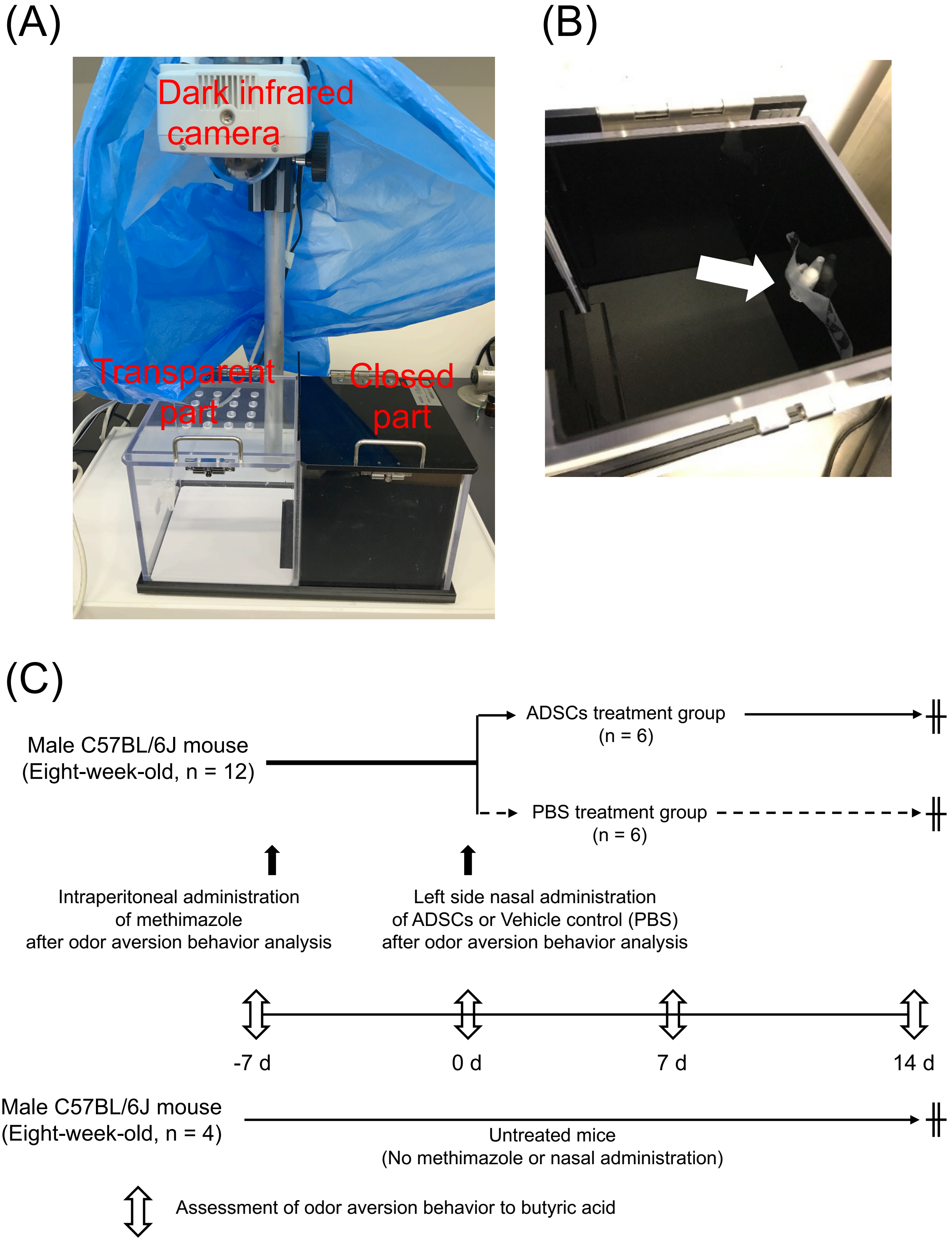

Figure S1. Odor aversion behavior analysis of mice treated with murine adipose-derived stem cells (ADSCs) .(A) Odor aversion behavior to butyric acid in mice was assessed in the transparent section of the testing box. (B) A cotton ball embedded with 300 μL 10% butyric acid was placed in an Eppendorf tube attached to the closed section of the box (arrow). (C) Experimental protocol: Twelve mice were intraperitoneally administrated with methimazole after the assessment of the odor aversion behavior to butyric acid 7 d before nasal administration of ADSCs (n = 6) or phosphate-buffered saline (PBS) (n = 6). The assessment of the odor aversion behavior to butyric acid was repeated on the day of nasal administration of ADSCs or PBS and after 7 and 14 days. ADSCs or PBS were nasally administrated into left side nasal cavity of each mouse under anesthesia as described in the methods after the assessment of the odor aversion behavior to butyric acid. The assessment of odor aversion behavior to butyric acid was repeated every 7 days in the four mice without any administration of methimazole, ADSCs or PBS.

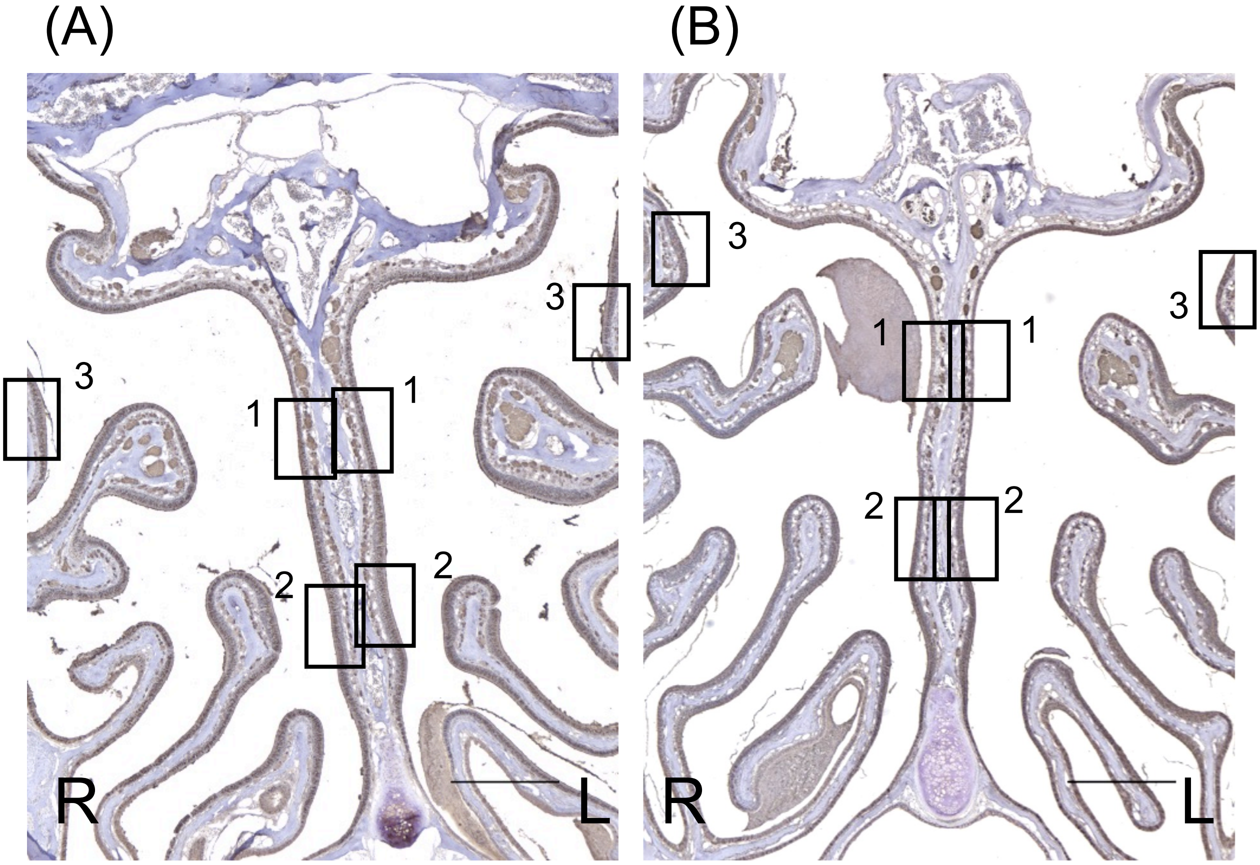

Figure S2. Immunohistochemical analysis of olfactory marker protein (OMP) expressions on the nasal septum and turbinates of mice 14 d after left side nasal administration of ADSCs or vehicle control (PBS). (A) ADSCs. (B) Vehicle control (PBS). Squares indicate the parts of an assessment in the lower power field image. Bars = 500 μm. R: Right, L: Left.

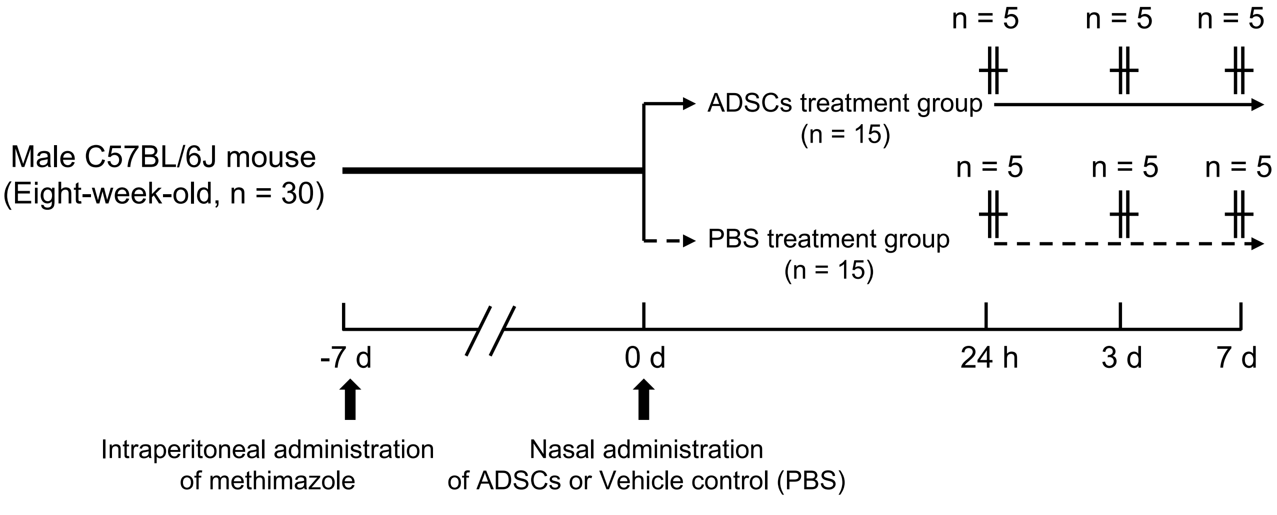

Figure S3. Experimental protocol: Immunohistochemical analysis of mice 24 h, 3 d, and 7 d after left side nasal administration of ADSCs or vehicle control (PBS). Thirty mice were nasally administrated with ADSCs (n = 15) or PBS (n = 15) 7 d after intraperitoneal administration of methimazole. The mice were perfused with physiological saline and fixed with 4% paraformaldehyde under anesthesia 24 h, 3 d, and 7 d after nasal administration of ADSCs or PBS.

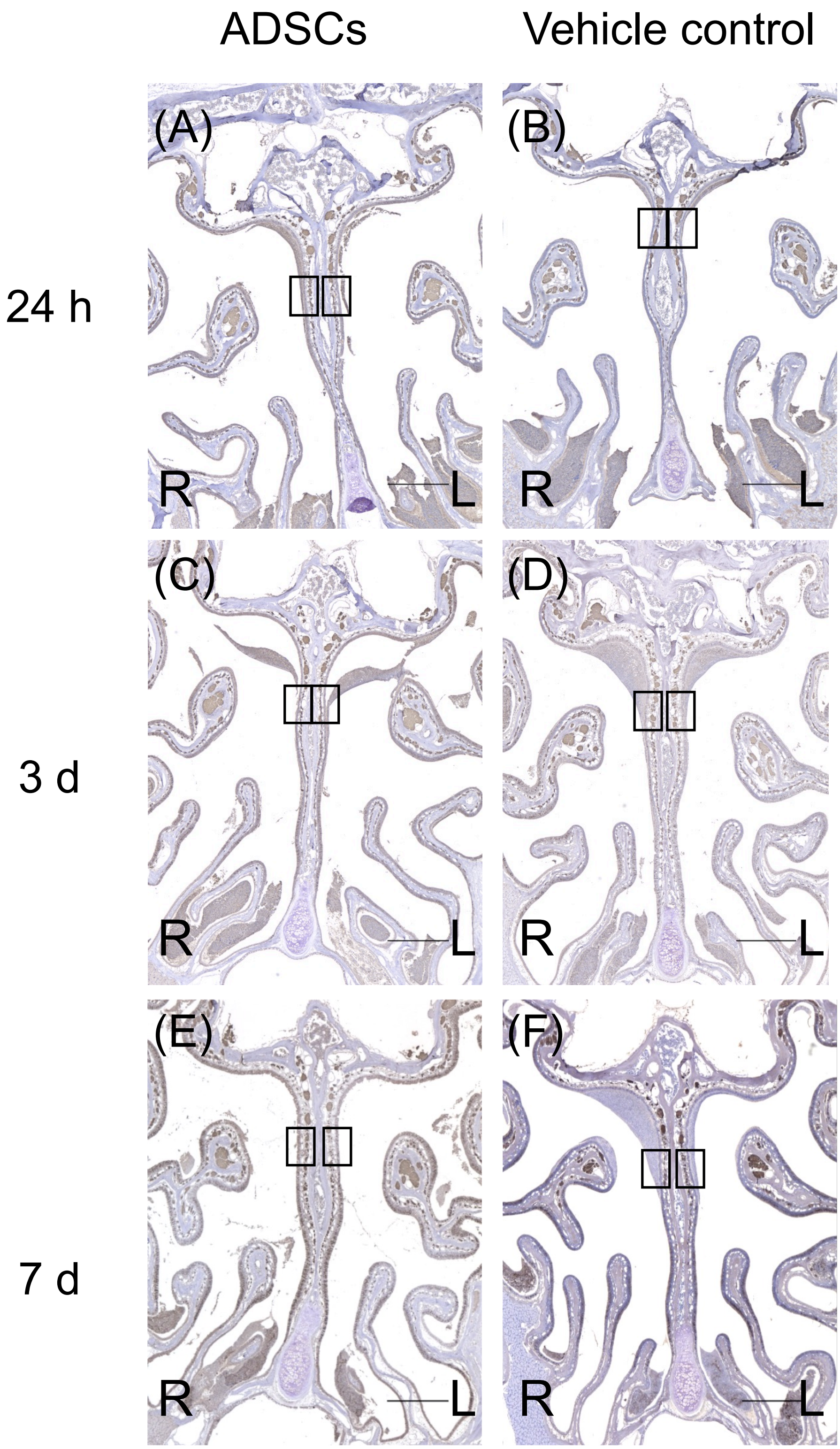

Figure S4. Immunohistochemical analysis of olfactory marker protein on the upper-middle region of the nasal septum of mice 24 h, 3 d, and 7 d after left side nasal administration of ADSCs or vehicle control (PBS). (A-F) Representative images of olfactory marker protein expression of five mice from each group, (A) 24 h, ADSCs (B) 24 h, PBS, (C) 3 d, ADSCs, (D) 3 d, PBS, (E) 7 d, ADSCs, (F) 7 d, PBS in mice. Squares indicate the parts of an assessment in the lower power field image. Bars = 500 μm. R: Right, L: Left.

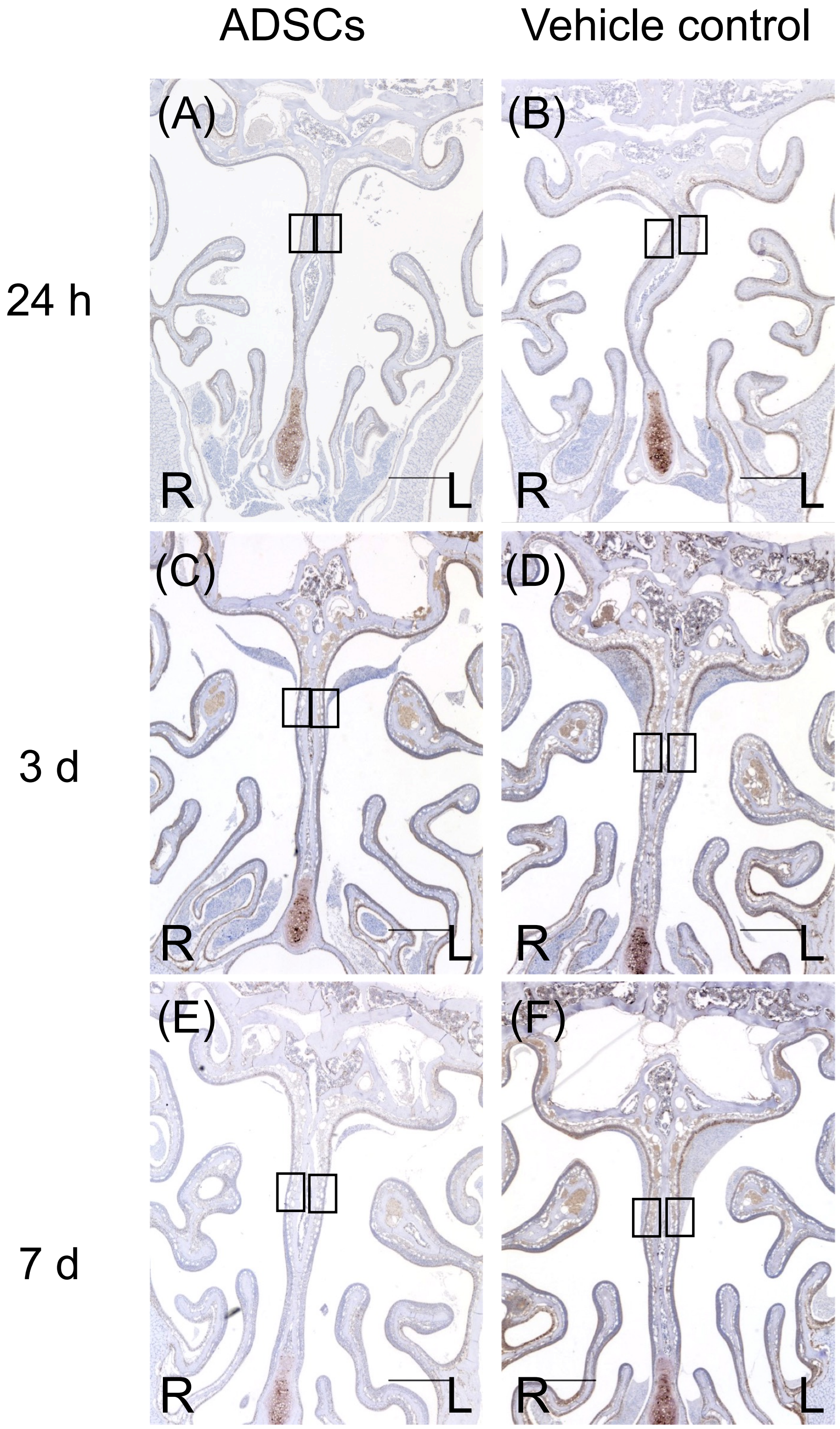

Figure S5. Immunohistochemical analysis of paired box 6 on the upper-middle region of the nasal septum of mice 24 h, 3 d, and 7 d after left side nasal administration of ADSCs or vehicle control (PBS); (A-F) Representative images of paired box 6 expression of five mice for each group, (A) 24 h, ADSCs (B) 24 h, PBS, (C) 3 d, ADSCs, (D) 3 d, PBS, (E) 7 d, ADSCs, (F) 7 d, PBS in mice. Squares indicate the parts of an assessment in the lower power field image. Bars = 500 μm. R: Right, L: Left.

3 d after nasal administration of ADSCs
Methimazole-treated mouse

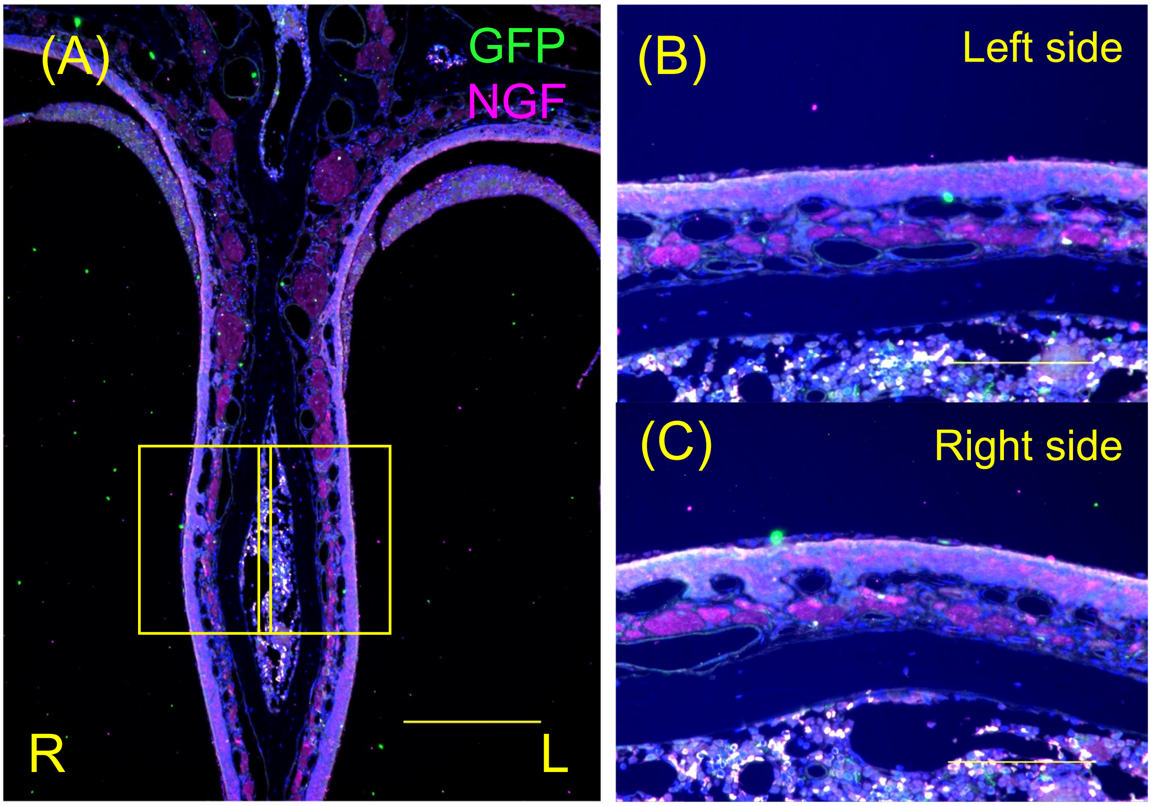

Figure S6. Immunohistochemical analysis of mice 3 d after left side nasal administration of ADSCs; Table S1: Primary antibodies used in this study; Table S2: Fluorescent secondary antibodies used in this study. Representative image of GFP (Green) and NGF (Purple) expression levels. Images are representative of five mice. Bars = 250 μm, 100 μm for (A), and (B, C), respectively. R: Right, L: Left.

Table S1. Primary antibodies used in this study.

Antibody	Species	Dilution	Company	Catalog #	Antigen
OMP	Goat	1:10000	FUJIFILM Wako	019-22291	Rodent olfactory marker protein
PAX6	Rabbit	1: 1,000	Abcam	ab195045	Recombinant fragment
NGF	Rabbit	1:250	Abcam	ab52918	Synthetic peptide
GFP	Goat	1: 1,000	Abcam	ab5450	Recombinant full-length protein

Abbreviations: GFP: green fluorescent protein; NGF: nerve growth factor; OMP: Olfactory marker protein; PAX6: paired box 6

Table S2. Fluorescent secondary antibodies used in this study .

Antibody	Species	Dilution	Company	Catalog #	Conjugate
Rabbit IgG (H+L) Cross-Absorbed	Donkey	1:200	Thermo Fisher Scientific	A-31573	Alexa Fluor 647
Goat IgG (H+L) Cross-Absorbed	Donkey	1:200	Thermo Fisher Scientific	A-21432	Alexa Fluor 555
